# B-cell malignancies treated with targeted drugs and SARS-CoV-2 infection: A European Hematology Association Survey (EPICOVIDEHA)

**DOI:** 10.3389/fonc.2022.992137

**Published:** 2022-10-04

**Authors:** Maria Stefania Infante, Jon Salmanton-García, Ana Fernández-Cruz, Francesco Marchesi, Ozren Jaksic, Barbora Weinbergerová, Caroline Besson, Rafael F. Duarte, Federico Itri, Toni Valković, Tomáš Szotkovski, Alessandro Busca, Anna Guidetti, Andreas Glenthøj, Graham P. Collins, Valentina Bonuomo, Uluhan Sili, Guldane Cengiz Seval, Marina Machado, Raul Cordoba, Ola Blennow, Ghaith Abu-Zeinah, Sylvain Lamure, Austin Kulasekararaj, Iker Falces-Romero, Chiara Cattaneo, Jaap Van Doesum, Klára Piukovics, Ali S. Omrani, Gabriele Magliano, Marie-Pierre Ledoux, Cristina de Ramon, Alba Cabirta, Luisa Verga, Alberto López-García, Maria Gomes Da Silva, Zlate Stojanoski, Stef Meers, Tobias Lahmer, Sonia Martín-Pérez, Julio Dávila-Vals, Jens Van Praet, Michail Samarkos, Yavuz M. Bilgin, Linda Katharina Karlsson, Josip Batinić, Anna Nordlander, Martin Schönlein, Martin Hoenigl, Zdeněk Ráčil, Miloš Mladenović, Michaela Hanakova, Giovanni Paolo Maria Zambrotta, Nick De Jonge, Tatjana Adžić-Vukičević, Raquel Nunes-Rodrigues, Lucia Prezioso, Milan Navrátil, Monia Marchetti, Annarosa Cuccaro, Maria Calbacho, Antonio Giordano, Oliver A. Cornely, José-Ángel Hernández-Rivas, Livio Pagano

**Affiliations:** ^1^ Hematology Deparment, Hospital Universitario Infanta Leonor, Madrid, Spain>; ^2^ Faculty of Medicine and University Hospital Cologne, Cologne Excellence Cluster on Cellular Stress Responses in Aging-Associated Diseases (CECAD), University of Cologne, Cologne, Germany; ^3^ Faculty of Medicine and University Hospital Cologne, Department I of Internal Medicine, Excellence Center for Medical Mycology (ECMM), University of Cologne, Cologne, Germany; ^4^ Hospital Universitario Puerta de Hierro, Majadahonda, Spain; ^5^ Hematology and Stem Cell Transplant Unit, IRCCS Regina Elena National Cancer Institute, Rome, Italy; ^6^ Department of Hematology, University Hospital Dubrava, Zagreb, Croatia; ^7^ Department of Internal Medicine, Hematology and Oncology, Masaryk University and University Hospital Brno, Brno, Czechia; ^8^ Centre Hospitalier de Versailles, Versailles, France; ^9^ San Luigi Gonzaga Hospital - Orbassano, Orbassano, Italy; ^10^ University Hospital Centre Rijeka, Rijeka, Croatia; ^11^ Croatian Cooperative Group for Hematological Diseases (CROHEM), Zagreb, Croatia; ^12^ Faculty of Medicine and Faculty of Health Studies University of Rijeka, Rijeka, Croatia; ^13^ University Hospital Olomouc, Olomouc, Czechia; ^14^ Stem Cell Transplant Center, AOU Citta’ della Salute e della Scienza, Turin, Italy; ^15^ Fondazione IRCCS Istituto Nazionale dei Tumori, Milan, Italy; ^16^ Department of Hematology, Copenhagen University Hospital - Rigshospitalet, Copenhagen, Denmark; ^17^ NIHR Oxford Biomedical Research Centre, Churchill Hospital, Oxford, United Kingdom; ^18^ Department of Medicine, Section of Hematology, University of Verona, Verona, Italy; ^19^ Department of Infectious Diseases, Karolinska University Hospital, Stockholm, Sweden; ^20^ Ankara University, Ankara, Turkey; ^21^ Clinical Microbiology and Infectious Diseases Department, Hospital General Universitario Gregorio Marañón, Madrid, Spain; ^22^ Health Research Institute IIS-FJD, Fundación Jimenez Diaz University Hospital, Madrid, Spain; ^23^ Division of Hematology and Oncology, Weill Cornell Medicine, New York, NY, United States; ^24^ Departement d’Hematologie Clinique, CHU de Montpellier, UMR-CNRS 5535, Universite de Montpellier, Montpellier, France; ^25^ King’s College Hospital, London, United Kingdom; ^26^ King’s College London, London, United Kingdom; ^27^ La Paz University Hospital, Madrid, Spain; ^28^ Hematology Unit, ASST-Spedali Civili, Brescia, Italy; ^29^ University Medical Center Groningen, Groningen, Netherlands; ^30^ Department of Internal Medicine, Albert Szent-Györgyi Health Center, Faculty of Medicine University of Szeged, Szeged, Hungary; ^31^ Communicable Disease Center, Hamad Medical Corporation, Doha, Qatar; ^32^ ASST Grande Ospedale Metropolitano Niguarda, Milan, Italy; ^33^ ICANS, Strasbourg, France; ^34^ Hematology Department, Hospital Universitario de Salamanca, Salamanca, Spain; ^35^ IBSAL, Centro de Investigación del Cáncer-IBMCC (USAL-CSIC), Salamanca, Spain; ^36^ Department of Hematology, Vall d’Hebron Hospital Universitari, Experimental Hematology, Vall d’Hebron Institute of Oncology (VHIO), Vall d’Hebron Barcelona, Spain; ^37^ Hospital Campus, Barcelona, Spain; ^38^ Departament de Medicina, Universitat Autònoma de Barcelona, Bellaterra, Spain; ^39^ Azienda Ospedaliera San Gerardo - Monza, Monza, Italy; ^40^ Università Milano-Bicocca, Milan, Italy; ^41^ Health Research Institute IIS-FJD, Fundacion Jimenez Diaz University Hospital, Madrid, Spain; ^42^ Portuguese Institute of Oncology, Lisbon, Portugal; ^43^ University Clinic of Hematology, Skopje, North Macedonia; ^44^ AZ KLINA, Brasschaat, Belgium; ^45^ Medizinische Klinik II, Klinikum rechts der Isar, TU München, Munich, Germany; ^46^ Hospital Nuestra Señora de Sonsoles, Ávila, Spain; ^47^ Department of Nephrology and Infectious Diseases, AZ Sint-Jan Brugge-Oostende AV, Brugge, Belgium; ^48^ Laikon General Hospital, Athens, Greece; ^49^ ADRZ, Goes, Netherlands; ^50^ UHC Zagreb, Zagreb, Croatia; ^51^ Department of Oncology, Hematology and Bone Marrow Transplantation with Section of Pneumology, University Medical Center Hamburg-Eppendorf, Hamburg, Germany; ^52^ Division of Infectious Diseases and Global Public Health, Department of Medicine, University of California San Diego, San Diego, CA, United States; ^53^ Clinical and Translational Fungal-Working Group, University of California San Diego, La Jolla, CA, United States; ^54^ Division of Infectious Diseases, Department of Internal Medicine, Medical University of Graz, Graz, Austria; ^55^ Institute of Hematology and Blood Transfusion, Prague, Czechia; ^56^ COVID Hospital “Batajnica”, Belgrade, Serbia; ^57^ Amsterdam UMC, location VUmc, Amsterdam, Netherlands; ^58^ U.O. Ematologia e Centro Trapianti Midollo Osseo, Ospedale Maggiore, Parma, Italy; ^59^ University Hospital Ostrava, Ostrava, Czechia; ^60^ Hematology and BMT Unit, Azienda Ospedaliera Nazionale SS. Antonio e Biagio e Cesare Arrigo, Alessandria, Italy; ^61^ Hematology Unit, Center for Translational Medicine, Azienda USL Toscana NordOvest, Livorno, Italy; ^62^ Hematology Department, Hospital Universitario 12 de Octubre, Madrid, Spain; ^63^ Hematology Unit, Fondazione Policlinico Universitario Agostino Gemelli - IRCCS, Rome, Italy; ^64^ Hematology Unit, Università Cattolica del Sacro Cuore, Rome, Italy; ^65^ University of Cologne, Faculty of Medicine and University Hospital Cologne, Clinical Trials Centre Cologne (ZKS Köln), Cologne, Germany; ^66^ University of Cologne, Faculty of Medicine and University Hospital Cologne, Center for Molecular Medicine Cologne (CMMC), Cologne, Germany; ^67^ German Centre for Infection Research (DZIF), Partner Site Bonn-Cologne, Cologne, Germany

**Keywords:** SARS-CoV-2, targeted drugs, infection risk, immune system COVID19, lymphoproliferative diseases (LPD), chronic lymphocytic leukemia (CLL), non-Hodgkin lymphoma (NHL)

## Abstract

Patients with lymphoproliferative diseases (LPD) are vulnerable to severe acute respiratory syndrome coronavirus 2 (SARS-CoV-2) infection. Here, we describe and analyze the outcome of 366 adult patients with chronic lymphocytic leukemia (CLL) or non-Hodgkin Lymphoma (NHL) treated with targeted drugs and laboratory-confirmed COVID-19 diagnosed between February 2020 and January 2022. Median follow-up was 70.5 days (IQR 0-609). Most used targeted drugs were Bruton-kinase inhibitors (BKIs) (N= 201, 55%), anti-CD20 other than rituximab (N=61, 16%), BCL2 inhibitors (N=33, 9%) and lenalidomide (N=28, 8%).Only 16.2% of the patients were vaccinated with 2 or more doses of vaccine at the onset of COVID-19. Mortality was 24% (89/366) on day 30 and 36%(134/366) on the last day of follow-up. Age >75 years (p<0.001, HR 1.036), active malignancy (p<0.001, HR 2.215), severe COVID-19 (p=0.017, HR 2.270) and admission to ICU (p<0.001, HR 5.751) were risk factors for mortality at last day of follow up. There was no difference in OS rates in NHL vs CLL patients (p=0.306), nor in patients treated with or without BKIs (p=0.151). Mortality in ICU was 66% (CLL 61%, NHL 76%). Overall mortality rate decreased according to vaccination status, being 39% in unvaccinated patients, 32% and 26% in those having received one or two doses, respectively, and 20% in patients with a booster dose (p=0.245). Overall mortality rate dropped from 41% during the first semester of 2020 to 25% at the last semester of 2021. These results show increased severity and mortality from COVID-19 in LPDs patients treated with targeted drugs.

## Introduction

The coronavirus disease 2019 (COVID-19) pandemic has challenged particularly vulnerable individuals such as those with cancer (1–4). Even among patients with cancer, the overall outcome, degree of immunodeficiency, and effect of cancer therapy on immunocompetence vary widely, leading to very different outcomes, depending on the underlying malignancy and its treatment.

Lymphoproliferative diseases (LPD) are a group of malignancy associated with a marked immunodeficiency, characterized by hypogammaglobulinemia, qualitative and quantitative B- and T-cell defects (5), CD4+ lymphopenia, as well as innate immune dysfunction and neutropenia (6). These immunodeficiencies are a result of the disease itself and its treatment, and lead to impaired immune response to common pathogens and poor response to vaccination (7, 8)

The introduction of targeted agents in the treatment of B-cell malignancies has changed their management. These therapies attempt to harness power from the patient’s immune system to eradicate lymphoma. In chronic lymphoid leukemia (CLL), oral Bruton tyrosine-kinase inhibitors (BKIs) such as ibrutinib and acalabrutinib, and the BCL2 inhibitor (venetoclax) have been increasingly used, replacing conventional chemotherapy in frontline treatment because of their improved progression-free survival (9–13). In indolent lymphomas, several phosphoinositide 3-kinase (PIK3) inhibitors have been approved in patients with relapse disease (14–16), but the use of these agents has been limited due to toxicities, including infection. A combination of lenalidomide and rituximab is a safe and effective therapy for patients with refractory indolent lymphoma (17). Anti-CD30 and anti-PD1 have improved the prognosis of naïve (18) and relapsed (19) Hodgkin lymphomas, and the new antibody conjugate polatuzumab vedotin has been introduced in the treatment of diffuse large B-cell lymphoma (20).

Targeted drugs differ from conventional chemotherapy regarding the risk for infection. Opportunistic infections have been reported in patients receiving ibrutinib (21). Therapy with idelalisib has been associated with an overall risk of infection, especially fungal (16, 22, 23), and in combination with rituximab-bendamustine (RB), high rates of cytomegalovirus (CMV) reactivation have been reported (24). By contrast, venetoclax does not seem to be associated with additional risks of infection (23). The risk of infection in patients with LPD treated with brentuximab (25, 26) are variable while neutropenia is a common side effect.

Focusing on COVID-19 infection, several studies have reported impaired serologic response after COVID-19 vaccination in CLL patients, especially those treated with anti-CD20 antibodies in the 12 months prior to infection, followed by BKIs and venetoclax-treated patients (27–29).

Several small series of CLL patients with COVID-19, mostly treated with ibrutinib, have been published to date, reporting a high rate of mortality and severity of infection (30, 31). Ibrutinib was initially thought to improve the outcome of COVID-19, based on retrospective studies (32, 33), but several subsequent clinical trials failed to confirm such benefit (34).

To date, only limited data are available on the clinical course of COVID-19 in patients with different underlying LPD treated with targeted drugs. We undertook a retrospective international multicenter study to evaluate the outcome of COVID-19 in patients with LPD treated with targeted drugs and in order to identify potential predictors of outcome.

## Methods

In this retrospective observational, multicenter study, we collected data on adult patients with LPD who received targeted therapy and were diagnosed with COVID-19 between February 2020 and January 2022 across 25 countries that participated in the survey promoted by the European Hematology Association (EHA) – Scientific Working Group Infection in Hematology EPICOVIDEHA survey (35, 36).Targeted drugs included: BKIs (acalabrutinib, ibrutinib, zanabrutinib), BCL2 inhibitors (venetoclax), anti-CD20 antibodies (obinutuzumab, ofatumumab) anti-CD30 (brentuximab), anti-CD79 (polatuzumab), anti-PD1 (pembrolizumab, nivolumab), immunomodulatory drugs (IMiDs) (lenalidomide) and PI3K inhibitors (idelalisib). Patients treated with rituximab were not specifically included in this study as another analysis has been performed by the EPICOVIDEHA survey (35, 36). Confirmed cases of COVID-19 were defined by a positive reverse transcriptase-polymerase chain reaction (RT-PCR) assay of a specimen collected by a nasopharyngeal swab. Pandemic waves were defined in time as follows: January-June 2020 (n=108), July-December 2020 (n=144), January-June 2021 (n=62), July-January 2022 (n=52). Each institutional review board independently approved the study. The study was conducted following the Declaration of Helsinki. Researchers at each center collected data using an online questionnaire hosted at www.clinicalsurveys.net (EFS Fall 2018, Questback, Cologne, Germany). EPICOVIDEHA is registered at http://www.clinicaltrials.gov, with the identifier NCT 04733729. Only de-identified data were entered and analyzed. We obtained demographic data, comorbidities, and underlying hematological disease including clinically significant outcomes (hospital admission and intensive care unit [ICU] admission, vital status) and COVID-19 management strategies. The severity of COVID-19 at admission was graded according to the China Centers for Disease Control and Prevention definitions: mild (non-pneumonia and mild pneumonia), severe (dyspnea, respiratory frequency ≥30 breaths per min, SpO2 ≤93%, PaO2/FiO2 50%), and critical (respiratory failure, septic shock, or multiple organ dysfunction or failure).

SPSSv25.0 was used for statistical analyses (SPSS, IBM Corp., Chicago, IL, United States). Categorical variables were presented as frequencies and percentages, while continuous variables by the median, interquartile range (IQR), and absolute range. Additionally, overall mortality was evaluated by a Cox proportional hazard model. A Univariable Cox regression model was performed including variables that potentially played a role in the mortality of patients. Variables with a p-value ≤0.1 were considered for the multivariable analysis. The Multivariable Cox regression model was calculated by the Wald backward method, and only statistically significant variables were reported. A p-value ≤0.05 was considered statistically significant.

## Results

In the study period, we identified 366 patients with LPD receiving targeted drugs at diagnosis of COVID-19. The median age at COVID-19 diagnosis was 68 years (IQR 58-77, range 25-96).

### Characteristics of the cohort

**Figure 1:**
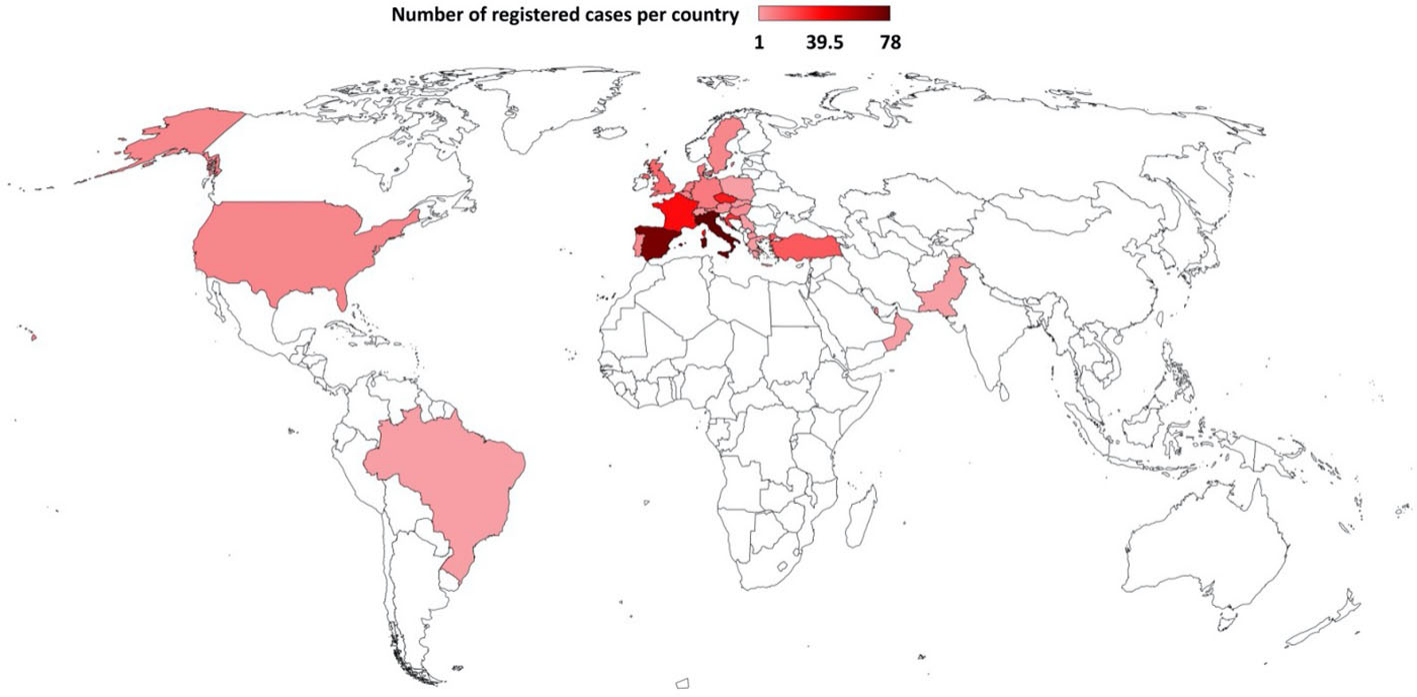
Countries contributing CLL and NHL cases to EPICOVIDEHA receiving targeted malignancy treatment, as of January 2022. Italy (n=78), Spain (n=72), France (n=37), Czech Republic (n=33), Croatia (n=27), Turkey (n=18), United Kingdom (n=14), Denmark (n=10), Germany (n=10), Netherlands (n=9), Belgium (n=8), Sweden and United States (n=7, each), Portugal (n=6), Austria, Hungary and Qatar (n=5, each), North Macedonia and Switzerland (n=3, each), Greece and Slovakia (n=2, each) and Brazil, Oman, Pakistan, Poland and Serbia (n=1, each).

Baseline characteristics for the entire cohort are described in **Table 1**. Of the 366 patients, 204 (55.7%) were CLL and 162 (44.3%) NHL. The population had a male predominance (n= 222, 60.7%) Contributing countries are listed in **Figure 1**. Around 33.1% (n= 132) of the patients had two or more comorbidities: chronic cardiopathy (n=125, 34.2%), chronic pulmonary disease (n=80, 21.9%) and diabetes (n= 64,17.5%) were the most common ones.

**Table 1:** Patients’ characteristics.

Sex		
Female	144	39.3%
Male	222	60.7%
**Age**, (IQR), [range]	68 (58-77) [25–96]	
18-25 years old	2	0.5%
26-50 years old	36	9.8%
51-69 years old	154	42.1%
≥ 70 years old	174	47.5%
**Comorbidities before COVID-19**		
No comorbidities	121	33.1%
1 comorbidity	113	30.9%
2 comorbidities	90	24.6%
3 or more comorbidities	42	11.5%
*Chronic cardiopathy*	125	34.2%
*Chronic pulmonary disease*	80	21.9%
*Diabetes*	64	17.5%
*Liver disease*	16	4.4%
*Renal impairment*	26	7.1%
*Obesity*	23	6.3%
*Smoking history*	43	11.7%
*No risk factor identified*	116	31.7%
**Malignancy**		
Chronic lymphoid leukemia	204	55.7%
Non-Hodgkin lymphoma	162	44.3%
**Malignancy status at COVID-19 diagnosis**		
Controlled malignancy	213	58.2%
*Complete remission*	92	25.1%
*Partial remission*	121	33.1%
Stable malignancy	59	16.1%
Active malignancy	84	23.0%
*Onset*	15	4.1%
*Refractory/Resistant*	69	18.9%
Unknown	10	2.7%
**Time last chemotherapy before COVID-19**		
In the last month	255	69.7%
In the last 3 months	52	14.2%
Chemotherapy ended > 3 months before COVID-19	57	15.6%
**# lines until COVID-19 onset**		
1 line	128	35.0%
2 lines	109	29.8%
3 lines	69	18.9%
4 lines	30	8.2%
>4 lines	30	8.2%
**Neutrophils at COVID-19 onset**		
≤ 500	15	4.1%
501 - 999	17	4.6%
≥ 1000	285	77.9%
**Lymphocytes at COVID-19 onset**		
≤ 200	22	6.0%
201 - 499	43	11.7%
≥ 500	249	68.0%
**Last vaccination before COVID-19**		
Not vaccinated	288	78.7%
One dose	19	5.2%
Two doses	54	14.8%
Three doses	5	1.4%
**Last type of vaccination before COVID-19**		
mRNA	65	17.8%
*BioNTech/Pfizer*	53	14.5%
*Moderna COVE*	12	3.3%
Vector-based	11	3.0%
*AstraZeneca Oxford*	9	2.5%
*Sputnik*	1	0.3%
*J&J - Janssen*	1	0.3%
Inactivated	2	0.5%
*CoronaVac | Sinovac*	2	0.5%
**COVID-19 severity**		
Asymptomatic	59	16.1%
Mild infection	53	14.5%
Severe infection	174	47.5%
Critical infection	80	21.9%
**COVID-19 symptoms at onset**		
Pulmonary	135	36.9%
Pulmonary + Extrapulmonary	107	29.2%
Extrapulmonary	62	16.9%
Screening	62	16.9%
**Stay during COVID-19 episode**		
Home	106	29.0%
Hospital	277	75.7%
*ICU*	80	21.9%
Invasive mechanical ventilation	55	15.0%
Non-invasive mechanical ventilation	29	7.9%
**Outcome**		
Mortality	134	36.6%
*COVID-19*	92	25.1%
*COVID-19 + Hematological malignancies*	28	7.7%
*Hematological malignancies ± other reason(s)*	14	3.8%

Thirty-five percent (n=128) of the patients were receiving the targeted drug as first-line therapy, 29.8% (n= 109) as 2^nd^ line, 18.9% (n=69) as 3^rd^ line, and 16.4% (n=60) had been heavily pretreated with 4 or more prior lines of therapy.

The most commonly used targeted drugs were BKIs (n=201,54.9%), anti-CD20 other than rituximab (n=60,16.4%), BCL2 inhibitors (n=33, 9%) and lenalidomide (n=28,7.7%) (**Table 2**). Of note, only 21.0% of the patients had received two or more doses of SARS-CoV-2 vaccine at the onset of COVID-19: mRNA vaccines were administered in 83% patients. Respiratory symptoms were present in 66.1% of patients (n= 242) while 16.9% (n= 62) presented with extrapulmonary symptoms. Of note, our series includes 16.9% (n=62) asymptomatic COVID-19 patients detected upon screening.

**Table 2:** LPD directed therapy at time of COVID-19 diagnosis.

**Anti-CD20 ± combination**	60	16.4%
Obinutuzumab	58	15.8%
Ofatumumab	1	0.3%
Obinutuzumab + Lenalidomide	1	0.3%
**Anti-CD30 ± combination**	16	4.4%
Brentuximab	15	4.1%
Brentuximab + Nivolumab	1	0.3%
**Anti-CD79**	5	1.4%
Polatuzumab	5	1.4%
**Anti-PD1**	3	0.8%
Nivolumab	1	0.3%
Pembrolizumab	2	0.5%
**BCL2 ± combination**	33	9.0%
Venetoclax	27	7.4%
Obinutuzumab + Venetoclax	6	1.6%
**IMiDs**	28	7.7%
Lenalidomide	28	7.7%
**BTKs ± combination**	201	54.9%
Ibrutinib	172	47.0%
Acalabrutinib	6	1.6%
Zanabrutinib	8	2.2%
Ibrutinib + Obinutuzumab	1	0.3%
Ibrutinib + Venetoclax	9	2.5%
Ibrutinib + Acalabrutinib	1	0.3%
Idelalisib + Acalabrutinib	1	0.3%
Obinutuzumab + Zanabrutinib	1	0.3%
Venetoclax + Acalabrutinib	2	0.5%
**PI3K inhibitor**	17	4.6%
Idelalisib	17	4.6%
**Other treatment combinations**	3	0.8%
Ibrutinib + Obinutuzumab + Venetoclax	1	0.3%
Ibrutinib + Idelalisib + Venetoclax	1	0.3%
Obinutuzumab + Venetoclax + Acalabrutinib	1	0.3%

The majority of patients (n=277, 75.7%) were hospitalized, with a median stay of 16 days (IQR 8-26, range, 1-137).

### Factors associated with severe COVID-19

Severe COVID-19 was observed in 47.5% (n=174) of patients, including 21.9% (n=80) who were admitted to intensive care unit (ICU). Among the latter, 55 (68.8%) were CLL patients, and 25 (31.3%) were non-Hodgkin lymphoma (NHL) patients. Fifty-five (44%) of the ICU-admitted patients underwent invasive mechanical ventilation. The median ICU stay in the entire cohort was 9 days (IQR 2-50, range, 6-14).

The presence of comorbidities was significantly associated with severe COVID-19 infection in the entire cohort (p= 0.002) as well as in the CLL and NHL subsets and BKIs cohort. Severe infection was more frequent in the first COVID-19 pandemic wave comparing to more recent waves (p=0.001). Another factor associated with severe infection was male sex (p=0.001). Age (both >65 or >75), type of targeted drug therapy and time from the last treatment of the hematologic malignancy to COVID-19 infection were not associated with severe infection in any subgroup analysis. No significant risk factor for severe COVID-19 was found in patients receiving BLC-2 inhibitors plus anti-CD20 monoclonal antibodies.

### Factors associated with mortality

**Figure 2:**
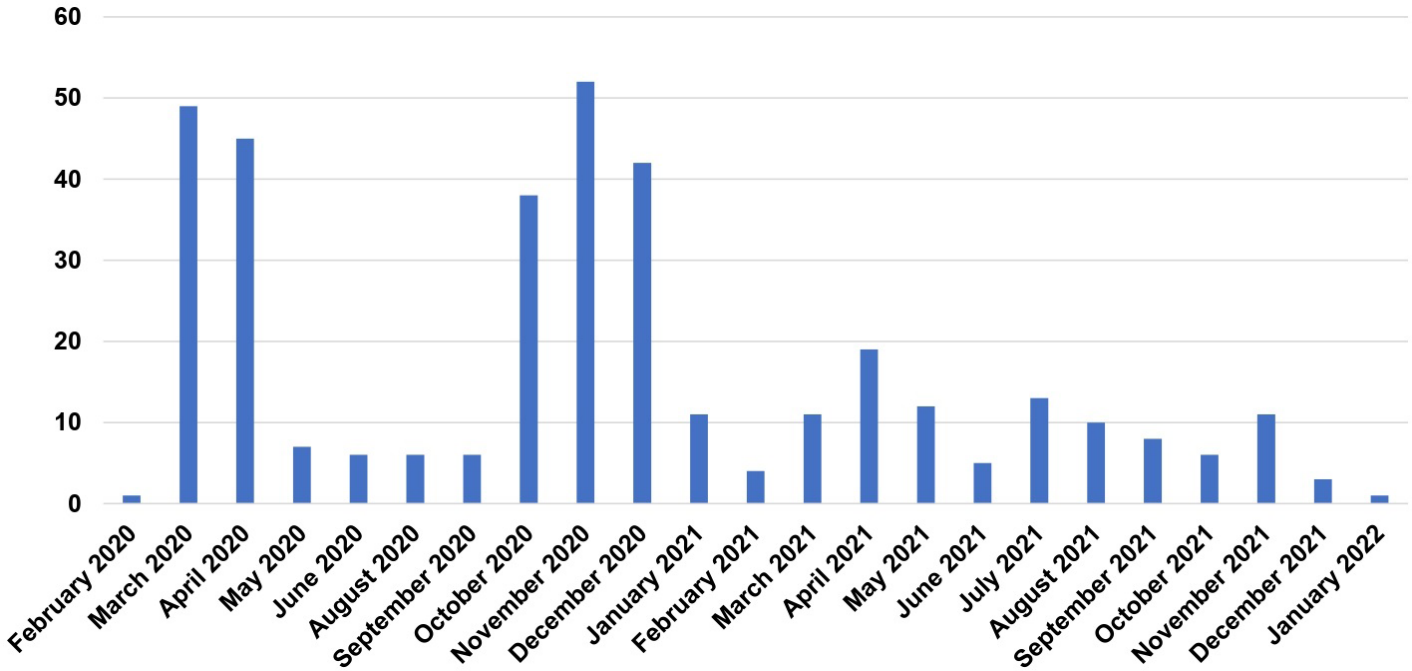
Distribution of registered cases along time.

Overall, 134 patients (36.6%) died (**Table 3**). The primary cause of death was COVID-19 in 92 patients (68.7%), LPD in 14 patients (10.4%), and a combination of both in 28 patients (20.9%). The mortality rate was 24.3% (89/366) on day 30 of COVID-19 diagnosis and 36.6% (134/366) on the last day of follow-up. The median follow-up at the time of this analysis was 70.5 days (IQR 19-159, range 0-609 days). Distribution of registered cases along time is shown in **Figure 2**.

**Table 3:** Patient’s disposition based on mortality.

	Alive		Dead		p value
	n	%	n	%	
**Sex**					0.294
Female	96	66.7%	48	33.3%	
Male	136	61.3%	86	38.7%	
**Age**					0.001
<65 years old	111	73.0%	41	27.0%	
≥65 years old	121	56.5%	93	43.5%	
**Comorbidities before COVID-19**					
Chronic cardiopathy	71	56.8%	54	43.2%	0.06
Chronic pulmonary disease	47	58.8%	33	41.3%	0.33
Diabetes	38	59.4%	26	40.6%	0.463
Liver disease	10	62.5%	6	37.5%	0.94
Renal impairment	7	26.9%	16	61.5%	<0.001
Obesity	18	78.3%	8	34.8%	0.521
Smoking history	24	55.8%	19	44.2%	0.272
No risk factor identified	79	68.1%	37	31.9%	0.202
**Time last chemotherapy before COVID-19**					0.658
In the last month	158	62.0%	97	38.0%	
In the last 3 months	33	63.5%	19	36.5%	
Chemotherapy ended > 3 months before COVID-19	39	68.4%	18	31.6%	
**Malignancy status at COVID-19 diagnosis**					0.003
Controlled disease	146	68.5%	67	31.5%	
Stable disease	40	67.8%	19	32.2%	
Active disease	40	47.6%	44	52.4%	

**Figure 3:**
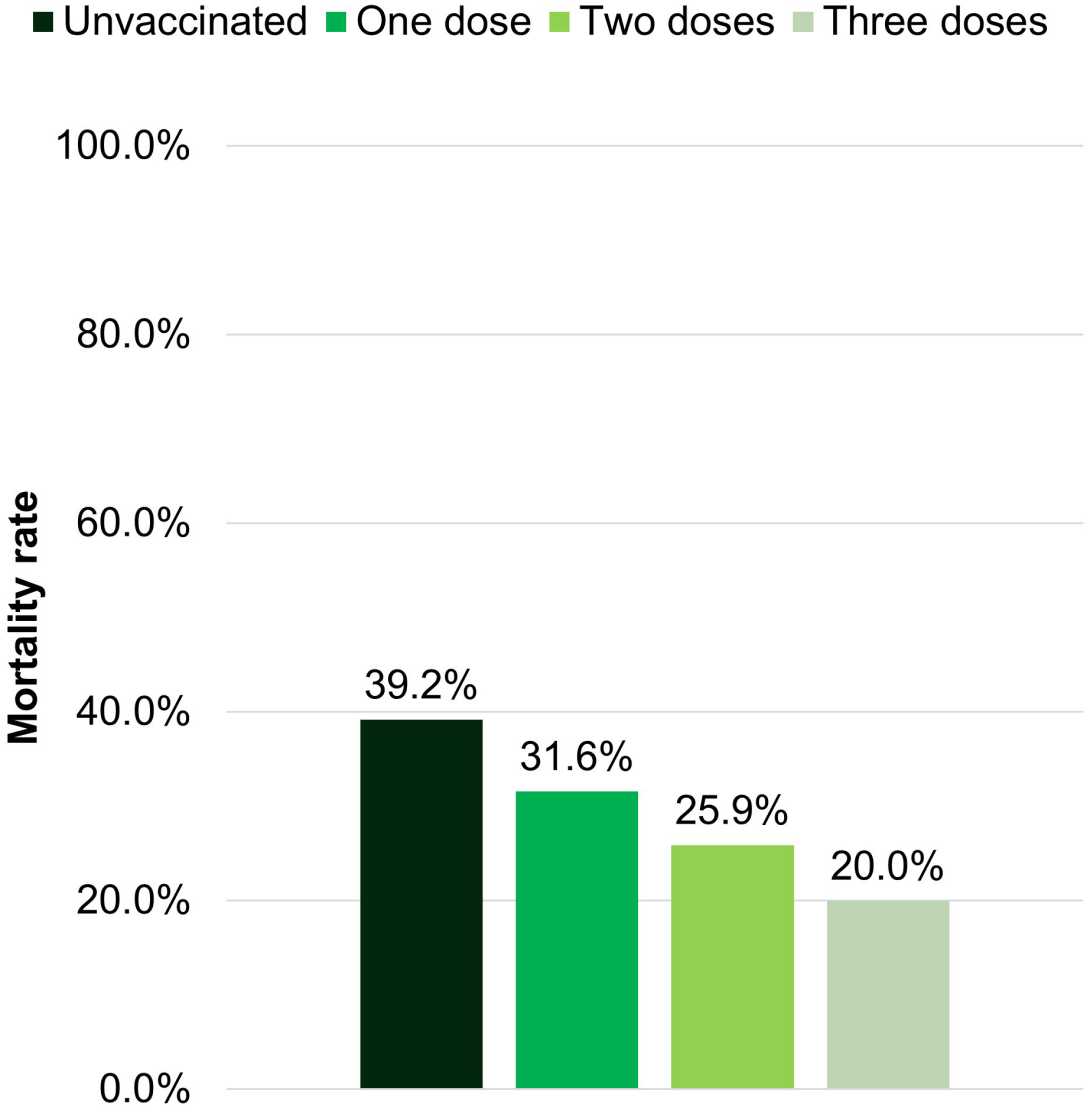
Mortality rate depending on the vaccination status.

Survival in patients admitted to ICU was 33.7% (CLL 38.1%, NHL 24%). The overall mortality rate decreased with vaccination, being 34.2% in unvaccinated patients, 15.9-18% with one or two doses, and 9.7% in patients with a booster dose (p<0.001) (**Figure 3**). Additionally, the mortality rate dropped from the first semester of 2020 (41.3%) to the last semester of 2021 (25%).

**Table 4A** summarizes the univariable and multivariable analyses of baseline characteristics as predictors of OS in the entire cohort and in the subsets of CLL and NHL patients (**Tables 4B**, **C** in supplementary materials). In univariable analysis, age >75 years, active hematological disease, severe and critical COVID-19 infection, heart disease, and renal impairment were associated with an increased mortality rate.

**Table 4A:** Univariable and multivariable analysis of predictors of mortality in the entire cohort.

	Univariable				Multivariable			
	p value	HR	95% CI		p value	HR	95% CI	
			Lower	Upper			Lower	Upper
**Sex**								
Female	-	-	-	-				
Male	0.336	1.191	0.835	1.698				
**Age**	<0.001	1.029	1.014	1.045	<0.001	1.036	1.019	1.052
**Status malignancy at COVID-19 diagnosis**								
Controlled malignancy	-	-	-	-	-	-	-	-
Stable malignancy	0.893	1.036	0.622	1.724	0.830	0.945	0.561	1.590
Active malignancy	0.003	1.798	1.222	2.646	<0.001	2.215	1.501	3.267
**Malignancy**								
Chronic lymphoid leukemia	-	-	-	-				
Non-Hodgkin lymphoma	0.330	1.185	0.842	1.669				
**COVID-19 infection**								
Asymtomatic	-	-	-	-	-	-	-	-
Mild	0.899	1.056	0.458	2.435	0.718	1.176	0.488	2.834
Severe	0.043	1.948	1.021	3.714	0.017	2.270	1.156	4.460
Critical	<0.001	4.563	2.376	8.764	<0.001	5.751	2.875	11.506
**Chronic cardiopathy**	0.025	1.491	1.052	2.113	0.775	1.056	0.726	1.536
**Chronic pulmonary disease**	0.393	1.188	0.800	1.763				
**Diabetes mellitus**	0.615	1.116	0.727	1.715				
**Liver disease**	0.837	1.090	0.480	2.474				
**Obesity**	0.820	0.920	0.450	1.882				
**Renal impairment**	<0.001	2.705	1.577	4.638	0.086	1.667	0.929	2.992
**Smoking history**	0.482	1.190	0.732	1.936				
**Neutrophils**								
≤ 500	-	-	-	-				
501 - 999	0.987	1.008	0.398	2.555				
≥ 1000	0.107	0.553	0.269	1.137				
**Lymphocytes**								
≤ 200	-	-	-	-				
201 - 499	0.874	1.067	0.479	2.375				
≥ 500	0.918	0.965	0.487	1.913				
**Time last chemotherapy**								
In the last month	-	-	-	-				
In the last 3 months	0.975	1.008	0.616	1.649				
>3 months before COVID-19	0.313	0.767	0.458	1.284				
**Chemotherapy lines before COVID-19**								
1	-	-	-	-				
2	0.062	1.516	0.979	2.347				
3	0.158	1.441	0.868	2.395				
4	0.043	1.886	1.020	3.489				
>4	0.061	1.867	0.972	3.584				
**SARS-CoV-2**								
α mutation (Alpha)	-	-	-	-				
β mutation (Beta)	0.837	1.121	0.376	3.343				
Wild type	0.953	1.032	0.367	2.902				
Not tested/Unknown	0.880	1.065	0.468	2.426				
**Vaccine doses before COVID-19**								
No vaccination	–	–	–	–				
One dose	0.972	1.015	0.445	2.312				
Two doses	0.829	0.940	0.537	1.646				
Three doses	0.867	0.845	0.118	6.068				
**Time last vaccination to COVID-19**								
≤14 days before COVID-19	-	-	-	-				
>14 days before COVID-19	0.732	1.190	0.439	3.222				
**COVID-19 treatment**								
No treatment	-	-	-	-				
Antivirals +/- corticosteroids +/- plasma	0.292	1.752	0.617	4.974				
Monoclonal antibodies +/- corticosteroids +/- plasma	0.659	1.623	0.189	13.925				
Antivirals + monoclonal antibodies +/- corticosteroids +/- plasma	0.544	1.444	0.440	4.740				
Corticosteroids	0.022	3.119	1.180	8.246				
Plasma +/- corticosteroids	0.632	0.591	0.069	5.063				
Unknown	0.198	1.810	0.734	4.460				

**Table 4B:** Univariable and multivariable analysis of predictors of mortality in the CLL patients.

	CLL							
	Univariable				Multivariable			
	p value	HR	95% CI		p value	HR	95% CI	
			Lower	Upper			Lower	Upper
**Sex**								
Female	-	-	-	-				
Male	0.381	1.262	0.750	2.124				
**Age**	0.010	1.031	1.008	1.056	0.002	1.040	1.015	1.066
**Status malignancy at COVID-19 diagnosis**								
Controlled malignancy	-	-	-	-				
Stable malignancy	0.977	1.009	0.548	1.859				
Active malignancy	0.146	1.594	0.850	2.989				
**Malignancy**								
Chronic lymphoid leukemia								
Non-Hodgkin lymphoma								
**COVID-19 infection**								
Asymtomatic	-	-	-	-	-	-	-	-
Mild	0.911	0.913	0.184	4.523	0.988	0.988	0.199	4.897
Severe	0.057	3.162	0.964	10.366	0.053	3.227	0.984	10.582
Critical	0.001	7.090	2.170	23.162	<0.001	8.251	2.516	27.062
**Chronic cardiopathy**	0.081	1.532	0.949	2.472	0.480	1.200	0.723	1.991
**Chronic pulmonary disease**	0.435	1.235	0.728	2.095				
**Diabetes mellitus**	0.704	0.890	0.486	1.627				
**Liver disease**	0.294	1.629	0.655	4.049				
**Obesity**	0.804	1.112	0.481	2.571				
**Renal impairment**	<0.001	3.310	1.690	6.486	0.123	1.770	0.857	3.653
**Smoking history**	0.885	1.049	0.550	1.999				
**Neutrophils**								
≤ 500	-	-	-	-				
501 - 999	0.563	0.655	0.156	2.747				
≥ 1000	0.133	0.409	0.127	1.313				
**Lymphocytes**								
≤ 200	–	–	–	–				
201 - 499	0.127	5.009	0.634	39.609				
≥ 500	0.333	2.660	0.367	19.271				
**Time last chemotherapy**								
In the last month	-	-	-	-				
In the last 3 months	0.512	0.736	0.295	1.839				
>3 months before COVID-19	0.608	0.832	0.411	1.683				
**Chemotherapy lines before COVID-19**								
1	-	-	-	-				
2	0.063	1.739	0.971	3.114				
3	0.618	1.202	0.583	2.476				
4	0.082	2.398	0.894	6.432				
>4	0.221	1.851	0.691	4.962				
**SARS-CoV-2**								
α mutation (Alpha)	-	-	-	-				
β mutation (Beta)	0.853	1.140	0.285	4.563				
Wild type	0.684	0.750	0.187	3.000				
Not tested/Unknown	0.523	0.718	0.260	1.982				
**Vaccine doses before COVID-19**								
No vaccination	-	-	-	-				
One dose	0.759	1.172	0.425	3.237				
Two doses	0.946	1.026	0.488	2.159				
Three doses	0.815	1.267	0.175	9.173				
**Time last vaccination to COVID-19**								
≤14 days before COVID-19	-	-	-	-				
>14 days before COVID-19	0.847	0.892	0.280	2.841				
**COVID-19 treatment**								
No treatment	-	-	-	-				
Antivirals +/- corticosteroids +/- plasma	0.510	1.736	0.337	8.950				
Monoclonal antibodies +/- corticosteroids +/- plasma	0.974	0.000	0.000	.				
Antivirals + monoclonal antibodies +/- corticosteroids +/- plasma	0.185	3.158	0.577	17.278				
Corticosteroids	0.033	4.983	1.139	21.810				
Plasma +/- corticosteroids	0.690	1.629	0.148	17.978				
Unknown	0.226	2.402	0.581	9.923				

**Table 4C:** Univariable and multivariable analysis of predictors of mortality in the NHL patients.

	NHL							
	Univariable				Multivariable			
	p value	HR	95% CI		p value	HR	95% CI	
			Lower	Upper			Lower	Upper
**Sex**								
Female	-	-	-	-				
Male	0.536	1.170	0.711	1.926				
**Age**	0.002	1.031	1.011	1.052	<0.001	1.045	1.023	1.067
**Status malignancy at COVID-19 diagnosis**								
Controlled disease	-	-	-	-	-	-	-	-
Stable disease	0.912	1.056	0.406	2.742	0.940	0.964	0.368	2.524
Active disease	0.017	1.885	1.121	3.170	0.001	2.363	1.391	4.016
**Malignancy**								
Chronic lymphoid leukemia								
Non-Hodgkin lymphoma								
**COVID-19 infection**								
Asymtomatic	-	-	-	-	-	-	-	-
Mild	0.589	1.311	0.491	3.499	0.352	1.623	0.585	4.503
Severe	0.269	1.559	0.710	3.424	0.111	1.977	0.855	4.572
Critical	0.002	3.817	1.666	8.744	<0.001	5.969	2.450	14.543
**Chronic cardiopathy**	0.106	1.522	0.915	2.534				
**Chronic pulmonary disease**	0.596	1.175	0.648	2.131				
**Diabetes mellitus**	0.158	1.554	0.843	2.862				
**Liver disease**	0.332	0.375	0.052	2.721				
**Obesity**	0.477	0.599	0.146	2.453				
**Renal impairment**	0.207	1.815	0.719	4.584				
**Smoking history**	0.175	1.676	0.795	3.530				
**Neutrophils**								
≤ 500	-	-	-	-				
501 - 999	0.374	1.761	0.505	6.135				
≥ 1000	0.560	0.760	0.301	1.916				
**Lymphocytes**								
≤ 200	–	–	–	–				
201 - 499	0.252	0.573	0.221	1.486				
≥ 500	0.669	0.848	0.398	1.806				
**Time last chemotherapy**								
In the last month	-	-	-	-				
In the last 3 months	0.662	1.145	0.623	2.104				
>3 months before COVID-19	0.357	0.700	0.328	1.495				
**Chemotherapy lines before COVID-19**								
1	-	-	-	-				
2	0.521	1.247	0.635	2.447				
3	0.147	1.702	0.830	3.487				
4	0.234	1.627	0.730	3.625				
>4	0.174	1.836	0.764	4.415				
**SARS-CoV-2**								
α mutation (Alpha)	-	-	-	-				
β mutation (Beta)	0.791	1.274	0.212	7.669				
Wild type	0.608	1.536	0.297	7.935				
Not tested/Unknown	0.420	1.791	0.435	7.375				
**Vaccine doses before COVID-19**								
No vaccination	-	-	-	-				
One dose	0.766	0.807	0.196	3.323				
Two doses	0.692	0.841	0.358	1.976				
Three doses	0.968	0.000	0.000	.				
**Time last vaccination to COVID-19**								
≤14 days before COVID-19	-	-	-	-				
>14 days before COVID-19	0.441	2.176	0.301	15.713				
**COVID-19 treatment**								
No treatment	-	-	-	-				
Antivirals +/- corticosteroids +/- plasma	0.312	2.014	0.519	7.818				
Monoclonal antibodies +/- corticosteroids +/- plasma	0.282	3.494	0.357	34.211				
Antivirals + monoclonal antibodies +/- corticosteroids +/- plasma	0.665	0.672	0.111	4.060				
Corticosteroids	0.309	2.032	0.519	7.960				
Plasma +/- corticosteroids	0.969	0.000	0.000	.				
Unknown	0.466	1.549	0.477	5.027				

By multivariable analysis, age >75 years (hazard ratio [HR] 1.036, 95% confidence interval [CI] 1.019-1.052, p<0.001), active hematological malignancy (HR 2.215, 95% CI 1.501-3.267, p<0.001), severe COVID-19 disease (HR 2.270, 95% CI 1.156-4.460 p=0.017) and critical COVID-19 disease (HR 5.751, 95% CI 2.875-11.506, p<0.001) remained as risk factors for mortality in the entire cohort. All factors remained significant for NHL, while in CLL patients all but active malignancy was significant.

**Figure 4:**
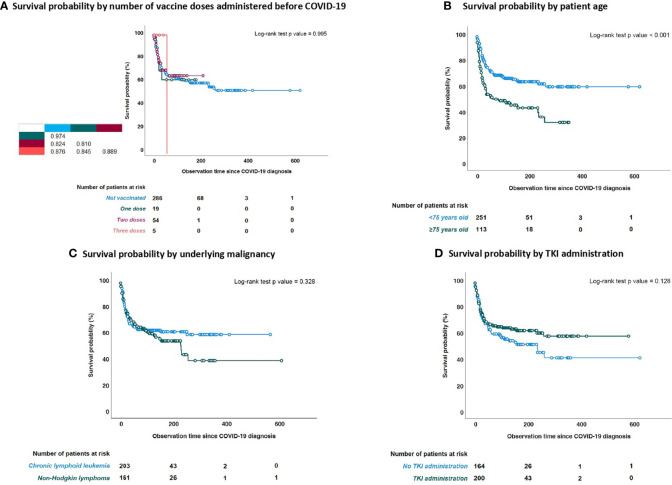
Survival probability (SP) from time of COVID-19 diagnosis stratified by number of vaccine doses, age, underlying malignancy and use of BKi at time of COVID-19 diagnosis. **(A)** SP stratified by number of vaccine doses administered; **(B)** SP stratified by age; **(C)** SP by underlying malignancy; **(D)** SP by BKi status.

There was no difference in OS in NHL vs CLL patients (p=0.344), in BKIs vs no BKIs-treated patients (p=0.137), nor when comparing patients treated with different targeted drugs (p=0.343) (**Figure 4**). We did not observe a clear protective or detrimental effect of BKIs on the outcome when compared with other targeted drugs.

## Discussion

To the best of our knowledge, we describe a large international series of LPD patients receiving targeted drug treatment at the time of COVID-19 infection. The rates of severe infection and overall mortality were 47.5% and 36.6%, respectively. The presence of comorbidities and lack of vaccination were associated with higher mortality rate. Prior vaccination was a protective factor. There were no significant differences in mortality across different targeted drugs. Patients treated with targeted chemotherapy were matched to controls treated with any other strategy for hematological malignancy before COVID-19. Cases and controls were matched in age, sex, hematological malignancy, malignancy status at COVID-19 and time of last chemotherapy strategy before COVID-19 (<3 months or >3 months). No statistically significant differences were observed in mortality probability between groups (p=0.056).

Patients with hematological malignancy have been heavily hit by the COVID-19 pandemic, and several reports confirm high rates of severe disease and mortality (2–4). Patients with B-malignancies have been particularly affected due to their intrinsic immune dysregulation (30, 31, 37–39). Moreover, the potential impact of LPD targeted therapies on the course of COVID-19 still needs to be fully understood.

The high mortality rates in our series appear similar to that of other series of hematological patients with COVID-19 infection (30, 31, 40–42). Surprisingly, despite the number of asymptomatic patients included in our study (diagnosed through screening for COVID-19) the rates of hospital admission and ICU admission were high. This data suggests that our cohort is at high risk of severe/critical COVID-19 when admitted to the hospital for symptomatic COVID-19.

Vaccination reduced mortality in our series, even after only 2 doses. Doubts have been raised about the efficacy of vaccination in patients with altered B cell immunity. Specifically in patients treated with anti-CD20, BKIs, or venetoclax, data demonstrating seroconversion failure after COVID-19 vaccination have been published (43, 44). Despite the lack of seroprevalence data in our series, we do consider that the COVID vaccines were a protective prognostic factor against mortality in these patients as mortality rates decreased as their vaccination status was increasing.

The mortality rate has decreased across the different COVID-19 waves, possibly reflecting improvements in patients care and the development of COVID-19 treatments: while we did not specifically examine COVID-19 treatment in our cohort, we can speculate that the early initiation of corticosteroids, heparin and the introduction of tocilizumab in the management of these patients might have improved the outcome. At the beginning of the pandemic, there was some reluctance about the use of corticosteroids and tocilizumab in hematological patients, due to the fear of stressing their immunodepression. Over time, the early initiation of those therapies was beneficial in those patients as well as the general population.

In our study, we did not find any association between specific targeted drugs and mortality. The majority of patients in our cohort were treated with BKIs. In the initial phase of the pandemic, some data suggested that BKIs could modulate the immune response to COVID-19 infection through blockade of inflammatory cytokines in the lungs, with a reduction of hyperinflammatory response (45, 46). The widespread use of early dexamethasone treatment in patients with severe COVID-19, based on the RECOVERY trial (47), achieved a more effective suppression of the host humoral response through the downregulation of proinflammatory cytokine production. We did not find significant differences in OS among patients treated or not with BKIs, independently from the time of the initiation of the drug, nor in the most prevalent cohorts of targeted patients after BKIs: BCL2-inhibitors and anti- CD20. In addition, due to the limited number of patients treated with other therapies, we cannot draw any conclusion about their role in this asset.

In the present series, age >75 years, severe and critical COVID-19 infection, and active hematological disease were independent predictors of mortality. This is consistent with recent data from the EPICOVIDEHA (36) survey that described, in addition to those, other risk factors for mortality such as chronic cardiac disease, liver disease, renal impairment, smoking history, and ICU stay in a cohort of patients with various hematological malignancies. Description of risk factors in hematological patients is of great importance to identify patients at high risk and implement rapidly prophylactic measures such as vaccination, masking, social distancing, and antiCOVID19 specific prevention and treatment.

Limitations of our study include its retrospective design, which implies dependence on the accuracy of medical records, and possible selection bias. The heterogeneity of underlying diseases and drug exposure could be another limitation, as a confounding factor for infection risk in this series. We could not perform a direct comparison between targeted drug-treated patients and chemotherapy patients as those groups would be too heterogeneous to compare.

Another limitation is the lack of patients from the latest waves infected with the delta and omicron variants and an analysis to determine if the new vaccine boosters can continue to reduce mortality in those patients. Specifically, patients treated with anti-CD20, BTKi, and BCL2-inhibitors were seroconversion failure after COVID-19 vaccination have been described, could be considered to receive early treatment with antivirals and monoclonal antibodies (48–50) or pre-exposure prophylaxis (51).

We acknowledge the potential underscoring of the real incidence of COVID-19 in this population, as we included asymptomatic patients with positive screening for COVID-19 while we must account for several asymptomatic patients not tested and therefore not diagnosed.

Our contribution is the largest international multicentric series of LPD patients under targeted drug treatment with COVID-19 infection, with a long follow-up, providing real-world evidence for increased severe disease and mortality from COVID-19 in patients with LPD treated with targeted drugs. Targeted drugs do not seem to have an impact on the survival of these patients. Efforts to prevent and aggressively manage COVID-19 should be focused on patients at a high risk of developing COVID-19 complications such as those older than 75 years, with comorbidities, especially heart disease, and active malignancy at COVID-19 onset. The importance of vaccination should be stressed, even in this population with humoral immunity impairment where it was a protective factor for mortality. New insights into the management of the infection throughout the pandemic and the development of COVID-19 treatments showed benefits in this particularly vulnerable population.

## Data availability statement

The original contributions presented in the study are included in the article/supplementary material. Further inquiries can be directed to the corresponding author.

## Ethics statement

The EPICOVIDEHA study has been approved by the local Institutional Review Board and Ethics Committee of the Fondazione Policlinico Universitario Agostino Gemelli— IRCCS, Università Cattolica del Sacro Cuore of Rome, Italy (Study ID: 3226). The corresponding local ethics committee of each participating institution may approve additionally the EPICOVIDEHA study when applicable. EPICOVIDEHA is registered at http://www.clinicaltrials.gov, with the identifier (NCT number): NCT 04733729. The patients/participants provided their written informed consent to participate in this study.

## Author contributions

MI, JS-G and AF-C contributed to the study conception and design. All authors contributed to data collection. Material preparation and analysis were performed by JS-G. The first draft of the manuscript was written by MI and JS-G and all authors commented on previous versions of the manuscript. All authors read and approved the final manuscript.

## Funding

EPICOVIDEHA has received funds from Optics COMMITTM (COVID-19 Unmet Medical Needs and Associated Research Extension) COVID-19 RFP program by GILEAD Science, United States (Project 2020-8223).

## Acknowledgments

We would like to thank the following: Pavel Žák Guillemette Fouquet, Francesca Farina, Fatih Demirkan, Laman Rahimli, Christian Bjørn Poulsen, Malgorzata Mikulska, Sandra Malak, Jorge Labrador, Moraima Jiḿenez, Stefanie Gräfe, Maria Chiara Tisi, Noemí Fernaandez, Ľuboš Drgona, Rui Bergantim, Laura Serrano, Jög Schubert, Giuseppe Sapienza, Juergen Prattes, Irati Ormazabal-Vélez, Marcio Nucci, Lisset Lorenzo De La Peña, Alexandra Serris, Carolina Garćia-Vidal, Nicola Fracchiolla, Nurettin Erben, Giulia Dragonetti, Roberta Di Blasi, Martin Cernan, Elena Busch, Monika M. Biernat, Murtadha Al-Khabori, Florian Reizine, Natasha Ali, Verena Petzer, Maria Merelli, Johan Maertens, Nina Khanna, Tomás-José González-López.

## Conflict of interest

The authors declare that the research was conducted in the absence of any commercial or financial relationships that could be construed as a potential conflict of interest of this work.

## Publisher’s note

All claims expressed in this article are solely those of the authors and do not necessarily represent those of their affiliated organizations, or those of the publisher, the editors and the reviewers. Any product that may be evaluated in this article, or claim that may be made by its manufacturer, is not guaranteed or endorsed by the publisher.

## Abbreviations

BKIs, Bruton tyrosine-kinase inhibitors; CLL, chronic lymphocytic leukemia; COVID19 Coronavirus disease 2019; CMV, cytomegalovirus; HR, hazard ratio; IMiDs, immunomodulatory drugs; ICU, intensive care unit; IQR, Interquartile Range; LPD, lymphoproliferative diseases; NHL, non-Hodgkin lymphoma; OS, overall survival; PIK3, phosphoinositide 3-kinase; RT-PCR, reverse transcriptase-polymerase chain reaction; SARS-CoV-2, severe acute respiratory syndrome coronavirus 2; RB, rituximab-bendamustine.

## References

[1] Guan W-J Ni Z-Y Hu Y Liang W-H Ou C-Q He J-X. Clinical characteristics of coronavirus disease 2019 in China. N Engl J Med (2020) 382:1708–20. doi: 10.1056/NEJMoa2002032 PMC7092819 32109013

[2] Vijenthira A Gong IY Fox TA Booth S Cook G Fattizzo B. Outcomes of patients with hematologic malignancies and COVID-19: a systematic review and meta-analysis of 3377 patients. Blood (2020) 136:2881–92. doi: 10.1182/blood.2020008824 PMC7746126 33113551

[3] Wood WA Neuberg DS Thompson JC Tallman MS Sekeres MA Sehn LH. Outcomes of patients with hematologic malignancies and COVID-19: a report from the ASH research collaborative data hub. Blood Adv (2020) 4:5966–75. doi: 10.1182/bloodadvances.2020003170 PMC7724912 33278301

[4] Yigenoglu TN Ata N Altuntas F Bascı S Dal MS Korkmaz S. The outcome of COVID-19 in patients with hematological malignancy. J Med Virol (2021) 93:1099–104. doi: 10.1002/jmv.26404 PMC7436524 32776581

[5] Moreira J Rabe KG Cerhan JR Kay NE Wilson JW Call TG. Infectious complications among individuals with clinical monoclonal b-cell lymphocytosis (MBL): a cohort study of newly diagnosed cases compared to controls. Leukemia (2013) 27:136–41. doi: 10.1038/leu.2012.187 22781591

[6] Law N Taplitz RA. How I manage infection risk and prevention in patients with lymphoid cancer. Blood (2022) 139:1517–28. doi: 10.1182/blood.2019003687 34748625

[7] Ochoa-Grullón J Peña Cortijo A Guevara-Hoyer K Jiménez García C de la Fuente E de la Peña AR. B-cell haematological malignancies and SARS-CoV-2 infection: Could immunological interventions influence the outcome? EJHaem (2021) 2(3):503–7. doi: 10.1002/jha2.249 34518828 PMC8426868

[8] Perry C Luttwak E Balaban R Shefer G Morales MM Aharon A. Efficacy of the BNT162b2 mRNA COVID-19 vaccine in patients with b-cell non-Hodgkin lymphoma. Blood Adv (2021) 5:3053–61. doi: 10.1182/bloodadvances.2021005094 PMC8362658 34387648

[9] Burger JA Tedeschi A Barr PM Robak T Owen C Ghia P. Ibrutinib as initial therapy for patients with chronic lymphocytic leukemia. N Engl J Med (2015) 373:2425–37. doi: 10.1056/NEJMoa1509388 PMC4722809 26639149

[10] Shanafelt TD Wang XV Kay NE Hanson CA O’Brien S Barrientos J. Ibrutinib-rituximab or chemoimmunotherapy for chronic lymphocytic leukemia. N Engl J Med (2019) 381:432–43. doi: 10.1056/NEJMoa1817073 PMC6908306 31365801

[11] Sharman JP Egyed M Jurczak W Skarbnik A Pagel JM Flinn IW. Acalabrutinib with or without obinutuzumab versus chlorambucil and obinutuzmab for treatment-naive chronic lymphocytic leukaemia (ELEVATE TN): a randomised, controlled, phase 3 trial. Lancet (2020) 395:1278–91. doi: 10.1016/S0140-6736(20)30262-2 PMC8151619 32305093

[12] Al-Sawaf O Zhang C Tandon M Sinha A Fink A-M Robrecht S. Venetoclax plus obinutuzumab versus chlorambucil plus obinutuzumab for previously untreated chronic lymphocytic leukaemia (CLL14): follow-up results from a multicentre, open-label, randomised, phase 3 trial. Lancet Oncol (2020) 21:1188–200. doi: 10.1016/S1470-2045(20)30443-5 32888452

[13] Hiddemann W Barbui AM Canales MA Cannell PK Collins GP Dürig J. Immunochemotherapy with obinutuzumab or rituximab for previously untreated follicular lymphoma in the GALLIUM study: Influence of chemotherapy on efficacy and safety. J Clin Oncol (2018) 36:2395–404. doi: 10.1200/JCO.2017.76.8960 29856692

[14] Dreyling M Santoro A Mollica L Leppä S Follows G Lenz G. Long-term safety and efficacy of the PI3K inhibitor copanlisib in patients with relapsed or refractory indolent lymphoma: 2-year follow-up of the CHRONOS-1 study. Am J Hematol (2020) 95:362–71. doi: 10.1002/ajh.25711 31868245

[15] Flinn IW Miller CB Ardeshna KM Tetreault S Assouline SE Mayer J. DYNAMO: A phase II study of duvelisib (IPI-145) in patients with refractory indolent non-Hodgkin lymphoma. J Clin Oncol (2019) 37:912–22. doi: 10.1200/JCO.18.00915 30742566

[16] Salles G Schuster SJ de Vos S Wagner-Johnston ND Viardot A Blum KA. Efficacy and safety of idelalisib in patients with relapsed, rituximab- and alkylating agent-refractory follicular lymphoma: a subgroup analysis of a phase 2 study. Haematologica (2017) 102:e156–9. doi: 10.3324/haematol.2016.151738 PMC5395130 27979923

[17] Morschhauser F Fowler NH Feugier P Bouabdallah R Tilly H Palomba ML. Rituximab plus lenalidomide in advanced untreated follicular lymphoma. N Engl J Med (2018) 379:934–47. doi: 10.1056/NEJMoa1805104 PMC11003525 30184451

[18] Straus DJ Długosz-Danecka M Alekseev S Illés Á Picardi M Lech-Maranda E. Brentuximab vedotin with chemotherapy for stage III/IV classical Hodgkin lymphoma: 3-year update of the ECHELON-1 study. Blood (2020) 135:735–42. doi: 10.1182/blood.2019003127 31945149

[19] Ansell SM Lesokhin AM Borrello I Halwani A Scott EC Gutierrez M. PD-1 blockade with nivolumab in relapsed or refractory hodgkin’s lymphoma. N Engl J Med (2015) 372:311–9. doi: 10.1056/NEJMoa1411087 PMC4348009 25482239

[20] Sehn LH Herrera AF Flowers CR Kamdar MK McMillan A Hertzberg M. Polatuzumab vedotin in relapsed or refractory diffuse Large b-cell lymphoma. J Clin Oncol (2020) 38:155–65. doi: 10.1200/JCO.19.00172 PMC7032881 31693429

[21] Rogers KA Mousa L Zhao Q Bhat SA Byrd JC Boghdadly ZE. Incidence of opportunistic infections during ibrutinib treatment for b-cell malignancies. Leukemia (2019) 33:2527–30. doi: 10.1038/s41375-019-0481-1 PMC7425823 31086260

[22] Zinzani PL Rambaldi A Gaidano G Girmenia C Marchetti M Pane F. Infection control in patients treated for chronic lymphocytic leukemia with ibrutinib or idelalisib: recommendations from Italian society of hematology. Leuk Res (2019) 81:88–94. doi: 10.1016/j.leukres.2019.04.016 31055248

[23] Teh BW Tam CS Handunnetti S Worth LJ Slavin MA. Infections in patients with chronic lymphocytic leukaemia: Mitigating risk in the era of targeted therapies. Blood Rev (2018) 32:499–507. doi: 10.1016/j.blre.2018.04.007 29709246

[24] Stefania Infante M Fernández-Cruz A Núñez L Carpio C Jiménez-Ubieto A López-Jiménez J. Severe infections in patients with lymphoproliferative diseases treated with new targeted drugs: A multicentric real-world study. Cancer Med (2021) 10(21):7629–40. doi: 10.1002/cam4.4293 34558211 PMC8559487

[25] Drgona L Gudiol C Lanini S Salzberger B Ippolito G Mikulska M. ESCMID study group for infections in compromised hosts (ESGICH) consensus document on the safety of targeted and biological therapies: an infectious diseases perspective (Agents targeting lymphoid or myeloid cells surface antigens [II]: CD22, CD30, CD33, CD38, CD40, SLAMF-7 and CCR4). Clin Microbiol Infect (2018) 24 Suppl 2:S83–94. doi: 10.1016/j.cmi.2018.03.022 29572070

[26] Connors JM Jurczak W Straus DJ Ansell SM Kim WS Gallamini A. Brentuximab vedotin with chemotherapy for stage III or IV hodgkin’s lymphoma. N Engl J Med (2018) 378:331–44. doi: 10.1056/NEJMoa1708984 PMC5819601 29224502

[27] Herishanu Y Rahav G Levi S Braester A Itchaki G Bairey O. Efficacy of a third BNT162b2 mRNA COVID-19 vaccine dose in patients with CLL who failed standard 2-dose vaccination. Blood (2022) 139:678–85. doi: 10.1182/blood.2021014085 PMC8648353 34861036

[28] Parry H McIlroy G Bruton R Ali M Stephens C Damery S. Antibody responses after first and second covid-19 vaccination in patients with chronic lymphocytic leukaemia. Blood Cancer J (2021) 11:136. doi: 10.1038/s41408-021-00528-x 34330895 PMC8323747

[29] Shen Y Freeman JA Holland J Solterbeck A Naidu K Soosapilla A. COVID-19 vaccine failure in chronic lymphocytic leukaemia and monoclonal b-lymphocytosis; humoural and cellular immunity. Br J Haematol (2021) 197(1):41–51. doi: 10.1111/bjh.18014 34962656

[30] Mato AR Roeker LE Lamanna N Allan JN Leslie L Pagel JM. Outcomes of COVID-19 in patients with CLL: a multicenter international experience. Blood (2020) 136:1134–43. doi: 10.1182/blood.2020006965 PMC7472711 32688395

[31] Scarfò L Chatzikonstantinou T Rigolin GM Quaresmini G Motta M Vitale C. COVID-19 severity and mortality in patients with chronic lymphocytic leukemia: a joint study by ERIC, the European research initiative on CLL, and CLL campus. Leukemia (2020) 34:2354–63. doi: 10.1038/s41375-020-0959-x PMC7347048 32647324

[32] Thibaud S Tremblay D Bhalla S Zimmerman B Sigel K Gabrilove J. Protective role of bruton tyrosine kinase inhibitors in patients with chronic lymphocytic leukaemia and COVID-19. Br J Haematol (2020) 190:e73–6. doi: 10.1111/bjh.16863 PMC7276870 32433778

[33] Treon SP Castillo JJ Skarbnik AP Soumerai JD Ghobrial IM Guerrera ML. The BTK inhibitor ibrutinib may protect against pulmonary injury in COVID-19-infected patients. Blood (2020) 135:1912–5. doi: 10.1182/blood.2020006288 PMC7243149 32302379

[34] Coutre SE Barnett C Osiyemi O Hoda D Ramgopal M Fort AC. Ibrutinib for hospitalized adults with severe COVID-19 infection: Results of the randomized, double-blind, placebo-controlled iNSPIRE study. Open Forum Infect Dis (2022) 9(5):ofac104. doi: 10.1093/ofid/ofac104 35493119 PMC8992313

[35] Salmanton-García J Busca A Cornely OA Corradini P Hoenigl M Klimko N. EPICOVIDEHA: A ready to use platform for epidemiological studies in hematological patients with COVID-19. Hemasphere (2021) 5:e612. doi: 10.1097/HS9.0000000000000612 34235404 PMC8232068

[36] Pagano L Salmanton-García J Marchesi F Busca A Corradini P Hoenigl M. COVID-19 infection in adult patients with hematological malignancies: a European hematology association survey (EPICOVIDEHA). J Hematol Oncol (2021) 14:168. doi: 10.1186/s13045-021-01177-0 34649563 PMC8515781

[37] Langerbeins P Eichhorst B. Immune dysfunction in patients with chronic lymphocytic leukemia and challenges during COVID-19 pandemic. Acta Haematol (2021) 144:508–18. doi: 10.1159/000514071 PMC8018219 33631756

[38] Roeker LE Eyre TA Thompson MC Lamanna N Coltoff AR Davids MS. COVID-19 in patients with CLL: improved survival outcomes and update on management strategies. Blood (2021) 138:1768–73. doi: 10.1182/blood.2021011841 PMC8313815 34297826

[39] Chatzikonstantinou T Kapetanakis A Scarfò L Karakatsoulis G Allsup D Cabrero AA. COVID-19 severity and mortality in patients with CLL: an update of the international ERIC and campus CLL study. Leukemia (2021) 35:3444–54. doi: 10.1038/s41375-021-01450-8 PMC8559135 34725454

[40] Infante M-S González-Gascón Y Marín I Muñoz-Novas C Churruca J Foncillas M-Á Landete E. COVID-19 in patients with hematological malignancies: A retrospective case series. Int J Lab Hematol (2020) 42:e256–9. doi: 10.1111/ijlh.13301 PMC7435496 32749757

[41] Malard F Genthon A Brissot E van de Wyngaert Z Marjanovic Z Ikhlef S. COVID-19 outcomes in patients with hematologic disease. Bone Marrow Transplant (2020) 55(11):2180–4. doi: 10.1038/s41409-020-0931-4 32376969 PMC7201203

[42] Martín-Moro F Marquet J Piris M Michael BM Sáez AJ Corona M. Survival study of hospitalized patients with concurrent covid-19 and haematological malignancies. Br J Haematol 190(1):e16–e20. doi: 10.1111/bjh.16801 PMC7267398 32379921

[43] Ghione P Gu JJ Attwood K Torka P Goel S Sundaram S. Impaired humoral responses to COVID-19 vaccination in patients with lymphoma receiving b-cell-directed therapies. Blood (2021) 138:811–4. doi: 10.1182/blood.2021012443 PMC8245303 34189565

[44] Passamonti F Romano A Salvini M Merli F Porta MGD Bruna R. COVID-19 elicits an impaired antibody response against SARS-CoV-2 in patients with haematological malignancies. Br J Haematol (2021) 195:371–7. doi: 10.1111/bjh.17704 PMC8444788 34272724

[45] Jacobs CF Eldering E Kater AP. Kinase inhibitors developed for treatment of hematologic malignancies: implications for immune modulation in COVID-19. Blood Adv (2021) 5:913–25. doi: 10.1182/bloodadvances.2020003768 PMC7871903 33560402

[46] Rezaei M Barati S Babamahmoodi A Dastan F Marjani M. The possible role of bruton tyrosine kinase inhibitors in the treatment of COVID-19: A review. Curr Ther Res Clin Exp (2022) 96:100658. doi: 10.1016/j.curtheres.2021.100658 34931090 PMC8673731

[47] The RECOVERY Collaborative Group. Dexamethasone in hospitalized patients with covid-19. N Engl J Med (2021) 384:693–704. doi: 10.1056/NEJMoa2021436 32678530 PMC7383595

[48] Cruz-Teran C Tiruthani K McSweeney M Ma A Pickles R Lai SK. Challenges and opportunities for antiviral monoclonal antibodies as COVID-19 therapy. Adv Drug Delivery Rev (2021) 169:100–17. doi: 10.1016/j.addr.2020.12.004 PMC7833882 33309815

[49] Zhang J Zhang H Sun L. Therapeutic antibodies for COVID-19: is a new age of IgM, IgA and bispecific antibodies coming? MAbs (2022) 14:2031483. doi: 10.1080/19420862.2022.2031483 35220888 PMC8890389

[50] Gaborit B Vanhove B Vibet M-A Le Thuaut A Lacombe K Dubee V. Evaluation of the safety and efficacy of XAV-19 in patients with COVID-19-induced moderate pneumonia: study protocol for a randomized, double-blinded, placebo-controlled phase 2 (2a and 2b) trial. Trials (2021) 22:199. doi: 10.1186/s13063-021-05132-9 33750432 PMC7942514

[51] O’Brien MP Forleo-Neto E Musser BJ Isa F Chan K-C Sarkar N. Subcutaneous REGEN-COV antibody combination to prevent covid-19. N Engl J Med (2021) 385:1184–95. doi: 10.1056/NEJMoa2109682 PMC8362593 34347950

